# Good sleep is a mood buffer for young women during menses

**DOI:** 10.1093/sleep/zsad072

**Published:** 2023-03-23

**Authors:** Alessandra E Shuster, Katharine C Simon, Jing Zhang, Negin Sattari, Andres Pena, Elisabet Alzueta, Massimiliano de Zambotti, Fiona C Baker, Sara C Mednick

**Affiliations:** Department of Cognitive Sciences, Sleep and Cognition Lab, University of California, Irvine, Irvine, CA, USA; Department of Cognitive Sciences, Sleep and Cognition Lab, University of California, Irvine, Irvine, CA, USA; Department of Cognitive Sciences, Sleep and Cognition Lab, University of California, Irvine, Irvine, CA, USA; Department of Cognitive Sciences, Sleep and Cognition Lab, University of California, Irvine, Irvine, CA, USA; Department of Cognitive Sciences, Sleep and Cognition Lab, University of California, Irvine, Irvine, CA, USA; Center for Health Sciences, SRI International, Menlo Park, CA, USA; Center for Health Sciences, SRI International, Menlo Park, CA, USA; Center for Health Sciences, SRI International, Menlo Park, CA, USA; Brain Function Research Group, School of Physiology, University of the Witwatersrand, Johannesburg, South Africa; Department of Cognitive Sciences, Sleep and Cognition Lab, University of California, Irvine, Irvine, CA, USA

**Keywords:** subjective sleep quality, menstrual cycle, women’s health, home testing, basic science, actigraphy, mental health

## Abstract

**Study Objectives:**

We sought to elucidate the interaction between sleep and mood considering menstrual cycle phase (menses and non-menses portions of the cycle) in 72 healthy young women (18–33 years) with natural, regular menstrual cycles and without menstrual-associated disorders. This work fills a gap in literature of examining mood in context of sleep and menstrual cycle jointly, rather than individually.

**Methods:**

Daily subjective measures of sleep and mood, and date of menses were remotely, digitally collected over a 2-month period. Each morning, participants rated their sleep on the previous night, and each evening participants rated the extent of positive and negative mood for that day. Objective sleep was tracked with a wearable (ŌURA ring) during month 2 of the study. Time-lag cross-correlation and mixed linear models were used to analyze the significance and directionality of the sleep–mood relationship, and how the interaction between menstrual cycle status and sleep impacted mood levels.

**Results:**

We found that menstrual status alone did not impact mood. However, subjective sleep quality and menstrual status interacted to impact positive mood (*p* < .05). After a night of perceived poor sleep quality, participants reported lower positive mood during menses compared to non-menses portions of the cycle, while after a night of perceived good sleep quality participants reported equivalent levels of positive mood across the cycle.

**Conclusions:**

We suggest that the perception of good sleep quality acts as a mood equalizer, with good sleep providing a protective buffer to positive mood across the menstrual cycle.

## Graphical Abstract

**Graphical Abstract:**
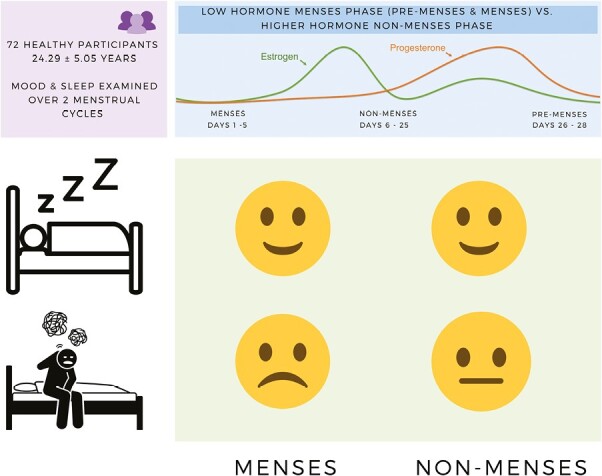

**Statement of Significance**

We found evidence that good sleep boosts positive mood regardless of menstrual cycle status, however poor sleep significantly lowers positive mood during menses more so than non-menses. These findings suggest that good sleep provides a protective buffer to positive mood during the menstrual cycle.

## Introduction

The menstrual cycle follows a roughly 28-day pattern, organized into phases marked by fluctuations in sex hormones including estrogen and progesterone among others [1]. The cycle starts with menses, marked by low levels of estrogen and progesterone across days 1–6 of the cycle. The levels of estrogen then rise, peaking on days 12–14, triggering ovulation (midcycle), dividing the menstrual cycle into the follicular and luteal phases. On days 15–28 (luteal phase), progesterone and estrogen remain high, before declining at the end of the luteal phase if implantation does not occur, and the next menses begins [2]. When examining the relation between mood and the menstrual cycle, studies reporting variability in mood across the cycle typically show that mood is worse right before and during menses compared to other phases of the cycle. Indeed, multiple studies using daily self-reported mood assessments over the course of at least one full menstrual cycle have reported that women have worse mood during the 3 days prior to menses and persisting through menses when hormone levels are low [3–8]. A more recent study looked at daily mood data and menstrual cycle status across 6 months. Although not all mood items showed variations across the cycle, this study did find increases for some, but not all, negative mood items during premenses and menses [9]. Taken together these studies indicate that, when an association between mood and menstrual cycle is present, it is strongest in the premenses and menses portions of the cycle.

Sleep is a factor that should be considered when examining menstrual cycle related variability in mood. Some research has found significant differences in self-reported sleep quality across the menstrual cycle, with poorer sleep just before and during menses even in young women without menstrual-associated complaints [10, 11]. Other research has provided evidence for a bidirectional relationship between sleep and the menstrual cycle, with average daily sleep duration impacting hormone concentrations and peak times, as well as menstrual phase impacting sleep duration [12]. However, not all women report a change in subjective sleep quality across the menstrual cycle, as shown in a study of 213 women who rated their nightly sleep across 2 months [13]. Cluster analysis revealed three patterns of subjective sleep and menstrual cycle status association; one group reported increased difficulty sleeping during the premenstrual phase, another showed a midcycle increase, while a third group reported no change in difficulty sleeping across the cycle. While subjective measures of sleep yield different results across studies and individuals, the majority of studies using objective measures of sleep efficiency via polysomnography and actigraphy or wearables indicate no variability across the cycle in healthy young adults [11, 14, 15].

Independent of the menstrual cycle, studies have shown a reciprocal relationship between sleep and mood [16]. Prior research provides evidence that low sleep quality predicts less positive mood and more negative mood [17, 18], and more positive mood leads to better sleep [19]. Two studies examined directionality of sleep and mood using subjective sleep and mood ratings and found a unidirectional relationship of subjective sleep predicting subsequent next-day mood [20, 21]. On the other hand, Talbot et al. showed directionality of mood impacting sleep using an experimental study with induction of positive mood via presentation of auditory and visual cues, finding that healthy participants had shorter reduced sleep onset latency (SOL) compared to a baseline with no induced mood in healthy adults, indicating a direction of mood impacting sleep [19]. Taken together, these studies suggest that sleep impacts mood, and mood may also impact sleep, however further work is needed to clarify the directionality of the sleep and mood relationship.

Here, we aimed to characterize the relationship between sleep and mood in healthy adult women, also considering the joint interaction effect between sleep and the menstrual cycle. We divided the menstrual cycle into two portions: menses (including three premenstrual days) and non-menses (all other days) due to previous findings associating mood with the menstrual cycle during this time. Based on prior literature, we predicted that women would report more negative and less positive moods during the menses phase compared with non-menses. Additionally, we expected that mood would be jointly impacted by the interaction between sleep and menstrual phase, with worst mood during menses after a poor night of sleep and best mood during non-menses after a good night of sleep. We expected to see this association for both subjective and objective measures of sleep.

## Methods

### Participants

This study included 76 healthy young female participants (see Table 1 for characteristics) who completed prescreening assessments to determine their sleep, menstrual, and general health status. Participants were recruited via campus flyers, social media ads, and mailers across the West Coast. Per social media and mailer metrics, upwards of 1000 people viewed our recruitment flyers. We conducted an a priori power analysis in G*Power using multiple linear regression which determined that *n* = 74 has 95% power to detect a moderate effect (Cohen’s *f*^2^ = 0.15). With anticipated attrition, we recruited a greater number of study participants than needed for adequate statistical power. We enrolled 111 participants, of which 35 withdrew, resulting in a final total of 76 participants.

**Table 1.** Participant characteristics

Characteristics		
Age (years)	*M* = 24.29	*SD* = 5.05

	N	% of total
Race/ethnicity		
Asian	32	42.11
White	23	30.26
Latino/Latina/Latinx	14	18.42
Black/African American	1	1.32
More than one race	6	7.89
Employment status^*^		
Unemployed	23	30.26
Part-time	24	31.58
Full-time	24	31.58
Not reported	5	6.58
BMI (kg m^−2^; self-reported)	*M* = 23.04	*SD* = 4.23
Menstrual cycle length (days)	*M* = 28.93	*SD* = 3.30

Characteristics of the 76 participants who completed the study. BMI, body mass index.

^*^A large proportion of the study sample came from a university population so many of those reporting unemployment are likely students, however we are unable to report exact numbers as we did not collect student status separately.

Participants had no current or past history of major psychological or medical conditions or sleep disorders, and additionally were not taking any medications (e.g. hypnotics) that would affect cognitive function or sleep. Participants completed the Beck Depression Inventory II (BDI-II) [22] and Generalized Anxiety Disorder 7 (GAD-7) [23] as screening assessments with a cutoff maximum of 10 on the GAD-7 and 20 on the BDI-II. All participants included in the study reported consistent sleep habits in advance of the study, and reported sleep times before 1 am and wake times before 10 am. Additionally, all participants had regular menstrual cycles with a consistent cycle length month-to-month (28.93 ± 3.3 days). None of the participants used hormonal contraceptives and none reported premenstrual symptoms as screened with the Premenstrual Syndrome Screening Tool [24].

### Procedure and assessment measures

**Figure 1.**
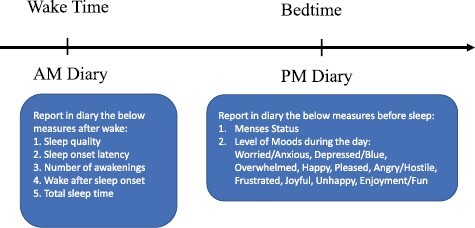
Sleep diary protocol: participants completed twice daily diaries across a 2-month time, answering questions about their subjective sleep the previous night in the morning and answering questions about their mood and menses status in the evening.

The study complied with the ethical standards of the relevant national and institutional committees on human experimentation and with the Helsinki Declaration of 1975, as revised in 2008, and was approved by the University of California, Irvine, IRB committee (HS# 2018-4652). All study participants completed an orientation session at the beginning of the study where they reviewed study details and processes. During this session study participants provided written informed consent and, upon completion, were enrolled in the study. Participants were instructed to continue their regular sleep schedule during the study and complete sleep diaries over a 2-month period, answering questions about their nighttime sleep, daily mood, and menstrual cycle (Figure 1). The sleep diaries were collected using a questionnaire in Qualtrics. Participants received email reminders each morning and evening with a link to the Qualtrics survey and were instructed to complete the diaries right before sleep and after waking up in the morning. Participants also wore an ŌURA ring for the second month of the study, which collected physiological sleep data.

### Subjective sleep measures

In the mornings, participants answered questions about their sleep the night before. The morning diary included the below measures of subjective sleep.

- *Sleep quality*
  Participants responded to the question “How was your sleep quality overall last night?” with response options on a scale of 1 (extremely bad) to 7 (extremely good).
- *SOL*
  To assess SOL participants responded to the question “How long did it take you to fall asleep last night?” on a sliding scale indicating the number of minutes.
- *Number of awakenings*
  Participants answered the question, “How many times did you wake up during the night?”
- *Wake after sleep onset (WASO)*
  To assess WASO participants were asked, “How many minutes were you awake during the night?”
- *Total sleep time (TST)*
  To assess TST participants responded to the question “How long were you asleep?” on a sliding scale indicating number of hours they were asleep.

### Mood status measures

In the evenings, participants completed a mood questionnaire within the sleep diary adapted from Sattari et al. [25]. Participants indicated the extent to which they felt Worried/Anxious, Depressed/Blue, Overwhelmed, Happy, Pleased, Angry/Hostile, Frustrated, Joyful, Unhappy, and Enjoyment/Fun throughout the day, on a 1–7 Likert scale. Response options were: Not at All, Very Slightly, Somewhat, Moderately, Much, Very Much, Extremely.

### Menstrual cycle measure

Each morning and evening participants were asked whether or not they started or were on their period that day. Response options consisted of: “Yes, I started my period today,” “Yes, I was on my period today,” and “No, I was not on my period today.” Evening answers were used as the daily data point for menses status. For instance, if a participant answered “No” in the morning, but “Yes” in the evening, that day’s data was treated as “Yes.”

### ŌURA ring

Participants wore ŌURA rings to collect physiological data during the second month of the study. The ŌURA ring is a sleep tracking device resembling a normal ring which collects physiological data from multiple sensors that rest on the palm-side of the finger. Participants were given a ring based on their estimated ring size and completed a setup session with a lab assistant to ensure the ring was appropriately set up and functioning. During this session participants downloaded the ŌURA application to their mobile device and created a depersonalized ŌURA account using their study participant identification number (SID). Upon getting a study participant’s consent, this SID ŌURA account was then connected the study’s ŌURA Cloud account. Participants were instructed to wear the ring at all times except for when charging the device or participating in activities which could damage the ring. The research team checked the ŌURA cloud data for each participant daily to ensure data were being collected and followed up with study participants if no data were recorded on a given day. ŌURA measures examined included: TST, SOL, and a disturbance score which ŌURA provides to represent sleep ­disturbances considering three measurement factors: (1) The number of times a participant woke up during the night; (2) The number of times a participant got up during the night; and (3) Amount of restless sleep as measured by motion detected during sleep. The ŌURA ring has been demonstrated to have high reliability for detecting sleep–wake accuracies, specificities, and sensitivities (between 0.88 and 0.89) [26].

### Data processing

All participants’ sleep diary responses were downloaded from Qualtrics and diary entries with insufficient information were excluded from further analysis. Exclusions were made for incomplete entries, which were determined if an entry was missing any of the following: menses status, mood status, or subjective sleep responses. Additional exclusions were made where either morning or night entry was completely missing. From the 2-month study period, an average of 3.64 days were excluded from the 2 months of diary entries per participant. Finally, one study participant was completely excluded for inconsistent diary entries and three study participants were excluded for missing or incomplete demographic and background surveys, leaving 72 study participants included in data analysis. The final dataset was prepared and analyzed in RStudio and MATLAB 2020a (MathWorks, Natick, MA).

All Likert scale responses were converted to a numeric scale. Scores for the question about sleep quality were categorized as either poor sleep (scores of 1–3) or good sleep (scores of 4–7). Categorizing these into two groups ensured that study participant variability in response did not affect results (i.e. one study participant with a poor night of sleep might respond “1” where another study participant with an equally poor night of sleep might respond “2”). Responses to the other subjective sleep questions were left as is since these were collected as continuous numeric data and not a Likert scale.

Three mood composites were created following the method of Romans et al. [9] by taking an average of combined individual mood items. Individual mood items were grouped into a Negative Mood Index (Anxiety, Depression, Overwhelmed, Unhappy), Positive Mood Index (Happy, Pleased, Joyful, Enjoyment/Fun), and an Anger/Frustration Index. Anger/Frustration was separated from the Negative Index as these items are not usually included in negative mood composites, which tend to focus on depressive/anxious moods [9, 27, 28].

ŌURA data were downloaded from the ŌURA cloud for each study participant and date matched to align with the study participant’s diary entries during the second month of the study. Exclusions were made for nights without any ŌURA sleep data recorded and data were cleaned to exclude data outside of three standard deviations from the mean per measurement, with an average of 2.86 nights of data excluded from the 1 month of ŌURA data collected per participant.

Finally, menses status was encoded as 0 for non-menses and 1 for menses. Menses included 3 days prior to start and through the end of menstruation. Non-menses included all remaining days of the cycle. Paired night and morning diary data entries were combined into one data entry line, consisting of menses status, sleep measures from a night of sleep, and mood the subsequent day.

### Data analysis models

First, we used a time-lag cross-correlation using xcorr in MATLAB between the time series of daily sleep measures and mood scores. Time-lag cross-correlation incrementally shifts one time series across the other, measuring the correlation at each shift point, and returns a peak correlation where the two time series have highest synchrony. This xcorr analysis was run individually between each mood composite (Positive, Negative, or Anger/Frustration) and each sleep measure (subjective sleep quality, SOL, number of awakenings, WASO, TST and objective TST, SOL, and sleep disturbance score). The process was run for each subject’s daily sleep and mood data, with the resulting peak in lag indicating directionality of sleep measure and mood. These metrics were then averaged across all study participants to determine the mean peak in lag across subjects. A statistically significant (*p* < .05) peak at lag = 0 indicates that no shift to data results in the strongest correlation, which means that prior sleep impacts next-day mood. Whereas lag = −1 indicates that the strongest correlation emerges when sleep is shifted left, aligning today’s mood with tonight’s sleep, indicating that mood leads sleep. A positive lag indicates that sleep potentially leads mood, but with a lag across multiple days.

Mixed linear models were then used to examine how daily mood varied as a function of daily menstrual phase (menses vs. non-menses) and sleep. Six models total were run using the lme function in R. Models were separated by the dependent factor of mood composite (Positive, Negative, or Anger/Frustration), with two models per composite. Each mood composite had one model with fixed factors of menstrual cycle status and subjective sleep measures (subjective sleep quality, SOL, number of awakenings, WASO, TST); and a second model with fixed factors of menstrual cycle status and objective sleep measures (ŌURA TST, SOL, sleep disturbance score). Separate models were created by objective and subjective sleep factors due to the collection of subjective measures across the entire study, while objective measures were collected for only part of the study. Per model we examined: the main effect of menstrual cycle, the main effect of each sleep factor, and the interaction effect between menstrual cycle and each sleep factor. We did not meet power requirements to expand our analysis to include potential confounding variables, which is noted as a limitation of this study.

## Results

### Time-lag cross-correlation between sleep and mood

Subjective sleep quality and positive mood were significantly correlated, with a mean peak in lag = 0 (*r* = 0.49, *p* < .05). We interpret lag = 0 as previous sleep predicting current mood as explained in the methods section. We did not find significant associations between other subjective/objective sleep measures and Positive Mood. We additionally did not find significant associations between any sleep metric and either the Negative or Anger/Frustration metrics.

### Relationship between mood, sleep, and menstrual cycle

**Figure 2.**
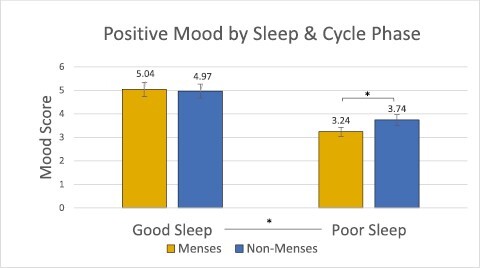
The positive mood composite by menstrual cycle phase (menses vs. non-menses) and sleep quality (good vs. poor). There was a significant main effect of sleep (*p* < .05) and a significant interaction effect between sleep and cycle status (*p* < .05) on positive mood. *marks significance of p < .05.

**Figure 3.**
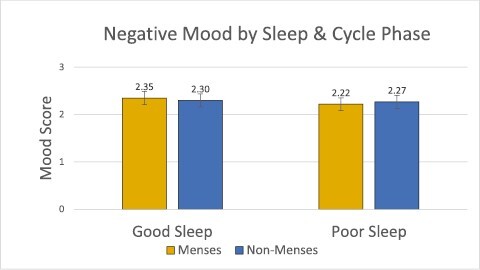
The negative mood composite by menstrual cycle phase (menses vs. non-menses) and sleep quality (good vs. poor). We found no significant evidence that cycle status, sleep, or the interaction between these impact negative mood (*p* > .05).

**Figure 4.**
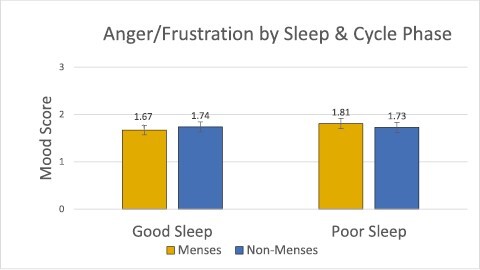
The anger/frustration composite by menstrual cycle phase (menses vs. non-menses) and sleep quality (good vs. poor). We found no significant evidence that cycle status, sleep, or the interaction between these impact anger/frustration (*p* > .05).

Results of the mixed linear models indicated no significant main effects of menstrual phase on mood (Positive Mood model: Cohen’s *f*^2^ = 0.05, *p* > .05; Negative Mood model: Cohen’s *f*^2^ = 0.008, *p* > .05; Anger/Frustration model: Cohen’s *f*^2^ = 0.02, *p* > .05). The mixed linear model examining the dependent variable of Positive Mood as a function of menstrual phase and subjective sleep factors showed a significant main effect of sleep quality on positive mood (Cohen’s *f*^2^ = 0.18, *p* < .05), with higher positive mood after a good night of sleep than a poor night of sleep. Additionally, in this model, the interaction between menstrual phase and sleep quality was significant (Cohen’s *f*^2^ = 0.16, *p* < .05, Figure 2). We conducted a post hoc power analysis in G*Power which determined that the final analysis size (*n* = 72) was sufficient to detect these results at *p* < .05, which is the statistical level referenced by the term significant throughout the rest of this manuscript. Post hoc comparisons revealed lower positive mood after a poor night of sleep during menses compared to non-menses (*t*(71) = 3.75, Cohen’s *d* = 0.81, *p* < .05, Figure 2), but no difference after a good night of sleep (*t*(71) = 0.58, Cohen’s *d* = 0.08, *p* > .05, Figure 2). To evaluate if an association between subjective sleep quality and menstrual phase existed, we ran a post hoc correlation analysis. Correlations were run between these two metrics per subject, and then the average correlation coefficient and *p* value were calculated. We found no significant association between subjective sleep quality and menstrual phase (*r* = −0.06, *p* > .05). See Table 2 for descriptive statistics of sleep measures across the menstrual cycle. In coherence with the time-lag cross-correlation results, no other subjective or objective sleep measure outside of subjective sleep quality had a significant main effect on positive mood, nor were there any interaction effects for the Negative and Anger/Frustration models (Figures 3 and 4).

**Table 2.** Sleep measures descriptive statistics

	Menses (*M* ± *SD*)	Non-menses (*M* ± *SD*)
Subjective (sleep diary)		
Sleep quality (score)	0.86 ± 0.14	0.89 ± 0.13
SOL (min)	15.9 ± 10.14	16.31 ± 11.19
Awakenings (#)	0.54 ± 0.63	0.55 ± 0.51
WASO (min)	5.80 ± 6.48	7.01 ± 7.87
TST (h)	7.74 ± 0.45	7.72 ± 0.40
Objective (ŌURA ring)		
SOL (min)	10.86 ± 3.25	10.70 ± 2.84
TST (h)	6.94 ± 0.45	6.86 ± 0.47
Sleep disturbances (score)	73.68 ± 7.42	73.48 ± 6.12

Descriptive statistics of subjective and objective sleep measures by cycle status.

## Discussion

The goal of the present study was to examine the interaction of menstrual cycle and sleep on mood in young women. We specifically compared the menses phase, including three premenstrual days, as compared to the remaining non-menses portion of the cycle, with self-reported measures of mood and sleep along with objective sleep measures provided by the ŌURA ring. Our results show that that while mood is not significantly predicted by menses status alone, menses status interacts with sleep to influence mood. A poor night of sleep results in lower mood during menses compared to non-menses. However, no difference was found in positive mood after a good night of sleep and no interaction with menstrual cycle. We did not find the same significance for negative mood metrics. These results suggest that positive mood is modulated jointly by sleep and menstrual cycle phase.

A woman’s menstrual cycle is often assumed to be associated with changes in mood, with menses characterized as a time of distress and negative mood increases [5]. However, our findings indicate that menses status alone does not significantly alter either positive or negative mood states in healthy, young women without menstrual-associated complaints. This finding is consistent with a review of mood and menstrual cycle literature which reported that, while some studies found evidence that negative mood increased during menses, most found no associations of mood and menstrual cycle [29]. Moreover, these findings are consistent with an earlier analysis of 26 study participants taken from the current study’s sample, which found no differences in an overall mood score (combined from positive, negative, and anger/frustration items) across the menstrual cycle without considering sleep as a factor in mood [15]. However, these findings may not be generalizable to those who experience premenstrual symptoms or with irregular cycles as these were exclusion screening factors for that analysis as well as the present study.

The menstrual cycle has also been associated with sleep, however we found no significant association between sleep and menstrual cycle phase (menses compared to non-menses). These findings also coincide with the earlier analysis of a subsample of the current study’s cohort [14].

However, some prior studies have found that participants reported lower sleep quality during the premenstrual and menses phases compared to the mid-follicular phase (reviewed in [11]), even in women without menstrual-associated complaints [10]. There are a few potential reasons for the discrepancy in results between studies. First, that study followed study participants over the course of one menstrual cycle, whereas the present study was over two menstrual cycles providing more robust data. Moreover, that study divided the menstrual cycle into five phases: premenstrual, menstrual, mid-follicular, periovulatory, and early/mid-luteal, whereas our study focused on a comparison of menses (including premenstrual days) with all remaining days (non-menses). Finally, a study of daily subjective sleep ratings across 2 months in a large group of 213 women found that not all women report a change in sleep quality across the menstrual cycle [13]. Cluster analysis revealed three patterns of subjective sleep and menstrual cycle status association with the largest cluster (46%) of women showing no change across the cycle but with smaller clusters showing either premenstrual (29%) or midcycle (25%) increases in difficulty sleeping. Moreover, increased anxiety and depression accounted for some of the variance in difficulty sleeping. Thus, it is possible that the present study found no changes across menses (including three premenstrual days) compared with non-menses due to screening out study participants with high depression, anxiety, or premenstrual symptoms.

Our time-lag cross-correlation analysis showed that subjective sleep quality influenced next-day mood, but daily mood did not influence that night’s sleep. This is consistent with prior research reporting a unidirectional relation of sleep leading mood [20, 21]. Our data suggest that there is a specific boost to positive mood following a good night of sleep. We did not replicate previously reported associations between subjective sleep quality and negative moods like sadness and depressive/anxious feelings [16, 18]. However, there are a growing number of studies showing that subjective sleep quality may be more strongly linked with positive mood states like happiness and joy [17, 30]. Moreover, our study focused on healthy, young women. It is possible that in women with clinical levels of psychiatric or premenstrual symptoms, subjective sleep quality could have next-day impacts to negative mood.

Other subjective sleep measures (SOL, number of awakenings, WASO, TST) and sleep physiological data collected via ŌURA ring did not significantly interact with the menstrual cycle to impact mood. It was, therefore, a more global measure of perceived sleep quality that interacted with mood across the menstrual cycle. Previous research has repeatedly shown discrepancies between measures of subjective and objective sleep [31–34], suggesting that objective and subjective sleep outcomes may reflect different aspects of sleep. On the other hand, we cannot exclude that performance limitations of the ŌURA ring [35] may have affected the results.

Prior studies rarely considered the directionality between sleep and mood in the context of the menstrual cycle. We found a significant interaction between subjective sleep quality and menstrual status, such that a night of poor sleep quality led to a lower positive mood during menses more so than non-menses, while there was no difference to positive mood after a good night of sleep. This finding suggests that a good night of sleep acts as a buffer to mood during menses.

### Limitations

This study had a few limitations. First, this study did not include data on hormones associated with the menstrual cycle, such as estrogen and progesterone, which excluded examination of how hormone variability across the cycle may contribute to mood, as well as our ability to identify menstrual phases by hormone level. Future work should collect biosamples such as blood or saliva to assess levels of estrogen and progesterone among other hormones to allow for additional phase-specific analysis across the menstrual cycle. Additionally, this study did not include polysomnography, the gold standard for sleep assessment, instead using subjective sleep measures and ŌURA measures. To fully understand sleep’s role in mood, future studies should include polysomnography to examine mechanisms in objective sleep which may be driving the results we see from subjective sleep. Additionally, while this study had a robust sample size to examine our variables of interest, we did not have sufficient power to include all potential confounding variables, such as age, race/ethnicity, or general health variables. Future studies should examine how these variables may impact the relation between sleep, the menstrual cycle, and mood. Finally, the current study included only young women who had no menstrual cycle complaints. Future research should expand to a wider age range, including older women who experience changes to their cycle as they approach menopause, as well as women who report menstrual cycle related mood disorders, like PMS, for whom sleep quality may be even more important as a buffer for positive mood in the context of menses. Moreover, important for future work is to examine daily changes in mood and sleep in male study participants to determine if male sex hormones influence the relation between sleep and mood.

### Summary

In conclusion, we found evidence that good sleep boosts positive mood, and that poor sleep significantly lowers positive mood during menses more so than during non-menses. These findings suggest that good sleep provides a protective buffer to positive mood during the menstrual cycle. Further studies are needed to identify and understand mechanisms that underlie the association between sleep and fluctuations in women’s sex hormones on daily mood.

## Data Availability

The data underlying this article will be shared on reasonable request to the corresponding author.
